# Impairment of Tricarboxylic Acid Cycle (TCA) Cycle in Alzheimer's Disease: Mechanisms, Implications, and Potential Therapies

**DOI:** 10.14336/AD.2025.0472

**Published:** 2025-05-29

**Authors:** Gudimetla Susmitha Mohan, Rahul Kumar

**Affiliations:** Department of Life Sciences, GITAM School of Sciences, GITAM (Deemed to be) University, Visakhapatnam, India

**Keywords:** Alzheimer's disease, TCA cycle, aconitase, isocitrate dehydrogenase, α-ketoglutarate dehydrogenase, succinate dehydrogenase

## Abstract

Alzheimer's disease (AD) is a neurodegenerative condition defined by the gradual impairment of cognitive functions, synaptic disarray, and extensive neuronal loss. Emerging evidence suggests that metabolic impairment, specifically within tricarboxylic acid (TCA) cycle, is instrumental in the AD pathophysiology. TCA cycle represents an indispensable pathway in metabolism that is responsible for energy production, and the maintenance of cellular homeostasis, particularly in neurons. Several *in vitro*, clinical, and *in vivo* studies reported that several TCA cycle enzymes disrupt during AD. Disruption in TCA cycle enzymes exhibits more pronounced impact on the brain owing to its high metabolic activity and continuous demand for energy, where any reduction in ATP production can severely impair neuronal function, synaptic plasticity, and overall cognitive processes. The current review explores the mechanisms underlying AD related impairment in TCA cycle, focussing on the molecular alterations of TCA enzymes. We also discussed potential activators and inhibitors of TCA cycle enzymes as a potential therapeutic intervention to restore AD related metabolic balance.

## 1. Introduction

Alzheimer's disease (AD) has been recognized as the primary cause of dementia in older adults, effecting around 44 million people worldwide [1], which is expected to increase by 3 folds after 2050 [2]. AD inflicts a substantial economic strain on the healthcare system, estimated at roughly 305 billion dollars in 2020 [3], and subsequently ranked as the third most expensive disease, following cancer and cardiovascular disease [4]. Continuous advancements in translational along with basic research have yielded significant insights into the underlying pathways; however, a comprehensive understanding remains elusive [5].

The amyloid hypothesis suggests that the accumulation of amyloid plaques is the initial event during AD pathogenesis [6], which is followed by neuronal death, synaptic dysfunction, neurofibrillary tangles (NFTs) formation and neuroinflammation [7]. Amyloid plaques primarily comprises of 36-43 amino acids long amyloid peptides (Aβ), which forms as a results of the proteolytic cleavage of amyloid precursor protein (APP) [8]. Notably, the sequential cleavage of APP by α-secretase and γ-secretase—a proteolytic complex with presenilin-1 as its core component—results in the generation of non-amyloidogenic fragments. In contrast, cleavage by β-secretase (specifically, beta-site amyloid precursor protein-cleaving enzyme 1, BACE-1) [9] followed by γ-secretase activity [10, 11] produces an amyloidogenic peptide that exhibits a propensity for spontaneous aggregation. Genetic studies further support this model by demonstrating that most mutations linked to AD risk are located within pathways governing APP processing [12-25]. Moreover, soluble Aβ oligomers are implicated in promoting synaptic dysfunction, neurodegeneration, and the hyperphosphorylation of tau at positions relevant to AD [26-28].

Another major hallmark of AD are intracellular aggregates referred to as NFTs which are primarily laden hyperphosphorylated tau-a microtubule-associated protein [29]. Under normal physiological conditions, tau interacts with tubulin to support microtubule assembly [30, 31]. Tau undergoes various post-translational modifications (PTMs), among which phosphorylation is the most intensively investigated, with around 85 potential phosphorylation sites identified [32-34]. It is well established that hyperphosphorylation of tau facilitates its aggregation whereas dephosphorylation tends to suppress self-assembly [35]. When tau becomes hyperphosphorylated, it loses its affinity for tubulin, leading to microtubule destabilization and neurotoxicity [36]. Additionally, the upregulation of tau-directed kinases exacerbates NFT formation by increasing tau phosphorylation [37-39].

Accumulation of reactive oxygen species (ROS) is recognised as a key contributor to the pathogenesis of AD as shown by various *in vitro* [40-43], *in vivo* [44-50] and clinical studies [51-64]. During AD, excessive ROS production, primarily due to the mitochondrial dysfunction [65-67], Aβ accumulation [68], and impaired antioxidant defences [69, 70], results in oxidative stress that damages vital cellular components including lipids, proteins, and nucleic acids [71-73]. Subsequently, oxidative stress disrupts neuronal metabolism along with the exacerbation of Aβ synthesis and tau hyperphosphorylation [74, 75]. Understanding the multifaceted role of ROS in AD opens avenues for exploring antioxidant therapies as potential neuroprotective strategies.

**Figure 1.**
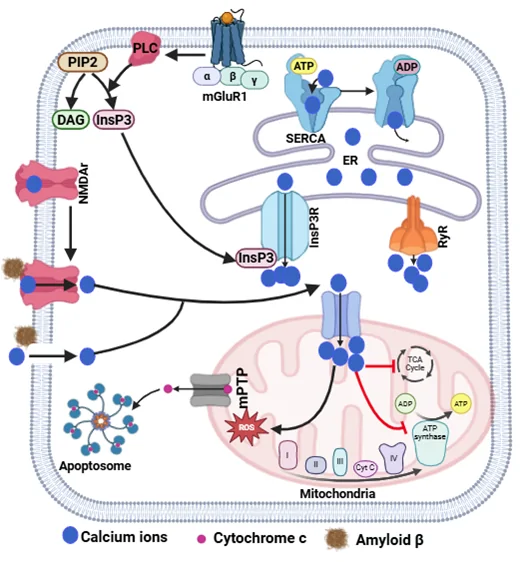
**Calcium dysregulation in Alzheimer’s Disease**. Binding of ligand to G protein coupled receptor mGluR1/5 results in the activation of phospholipase C, that in turn cleaves the phosphatidylinositol 4,5 bisphosphate (PIP2) into diacylglycerol and inositol 1,4,5 trisphosphate (InsP3). Subsequently, InsP3 stimulate the release of Ca^2+^ from endoplasmic reticulum (ER) into the cytosol via InsP3 receptor (InsP3R). The release of Ca2+ is further augment by ryanodine receptors (RyR) that is activated in response to an increase in the concentration of cytosolic Ca^2+^. On the other hand, Ca^2+^ influx into ER is mediated by sarcoplasmic and endoplasmic reticulum calcium ATPase (SERCA). Aβ promotes Ca^2+^ influx from extracellular environment via directly forming the pores in the neurons or through interaction with N-methyl-D-aspartate receptor (NMDARs). Accumulated Ca^2+^ is readily taken up by mitochondria with the help of mitochondrial Ca2+ uniporter (MCU) and leads to mitochondrial Ca^2+^ overload. The excessive accumulation of Ca2^+^ provokes a series of harmful events including augmented oxidative stress, ATP synthesis inhibition and activation of mitochondrial permeability transition pore (mPTP), which in turn facilitates the release of cytochrome C, formation of apoptosome and activation of apoptosis.

Cellular Ca^2+^ homeostasis plays a key role in regulating numerous aspects of neuronal physiology, including cellular growth, differentiation, action potential modulation, synaptic plasticity, and cognitive functions such as learning and memory [76]. Whereas, Conversely, perturbations in Ca^2+^ balance can trigger a cascade of deleterious events, including necrosis, apoptosis, impaired autophagic flux, and progressive neurodegeneration [77]. Endoplasmic reticulum is considered as the storehouse of Ca^2+^ and releases the cation upon the binding of binding the inositol 1,4,5 trisphosphate (InsP3) to the InsP3 receptor (InsP3R) [78-80]. InsP3 generation depends on the cleavage of phosphatidylinositol 4,5 bisphosphate via phospholipase C, that in turn is activated by the binding of glutamate to Glutamate stimulates activation of mGluR1/5 [81, 82]. The release of Ca^2+^ is further augment by ryanodine receptors (RyR) that is activated in response to an increase in the concentration of cytosolic Ca^2+^ [83]. On the other hand, Ca^2+^ influx into ER is mediated by sarcoplasmic and endoplasmic reticulum calcium ATPase (SERCA) [84]. An elevation in the intracellular Ca^2+^ was reported by Lopez and Colleagues using the neurons cultured from 3xTg-AD mice when compared with non-transgenic counterparts [85]. Several mechanisms have been identified that can results in the accumulation of Ca^2+^ including the formation of pores in the plasma membrane by Aβ oligomers, that specifically interacts with phosphatidylserine [85-87]. Other pathways includes the direct activation of N-methyl-D-aspartate receptor (NMDARs) by Aβ [86] and increased iron (Fe^2+^) and copper (Cu^+^) mediated lipid peroxidation in plasma membrane [88, 89]. Following their excessive accumulation, Ca^2+^ is actively taken up by mitochondria with the help of mitochondrial Ca^2+^ uniporter (MCU) and leads to mitochondrial Ca^2+^ overload [87]. The excessive accumulation of Ca^2+^ provokes a series of harmful events including augmented oxidative stress, ATP synthesis inhibition and activation of mitochondrial permeability transition pore (mPTP), which in turn facilitates the release of cytochrome C and activation of apoptosis [90] (Fig. 1).

Although the mammalian brain accounts for only about 2% of total body weight, it consumes roughly 20% of the body's oxygen to sustain its high metabolic demands [91]. Additionally, the brain demands a constant stream of glucose, which is processed through an interconnected network of metabolic pathways—namely glycolysis, the tricarboxylic acid cycle (TCA, also known as the Krebs or citric acid cycle), oxidative phosphorylation and electron transport chain (ETC) [92]. Within mitochondria, the TCA cycle converts acetyl CoA derived from glucose and fatty acids into high-energy molecules such as NADH, FADH_2_, and GTP. These molecules are critical for sustaining synaptic transmission, neurotransmitter synthesis, and the maintenance of membrane potential within neurons [93-95].

A critical interplay exists between the TCA cycle and Alzheimer’s disease pathology, where the accumulation of Aβ and tau hyperphosphorylation impairs TCA cycle function, resulting in ATP depletion that further promotes cellular demise [96]. Furthermore, the buildup of reactive oxygen species compromises key TCA cycle enzymes—especially those containing iron-sulfur clusters such as aconitase—leading to redox imbalances that exacerbate oxidative stress. Disruptions in the TCA cycle can significantly impair cerebral function by reducing ATP synthesis that hampers synaptic communications and contribute towards the accumulation of toxic metabolites. The current review focuses on how such disruptions influence AD pathophysiology, alongside exploring known inhibitors and activators of TCA cycle enzymes. These modulators have potential therapeutic implications, as targeting the regulation of key enzymes in the TCA cycle could offer new avenues for optimizing mitochondrial performance while decelerating AD progression.

## 2. Tricarboxylic acid (TCA) cycle

**Figure 2.**
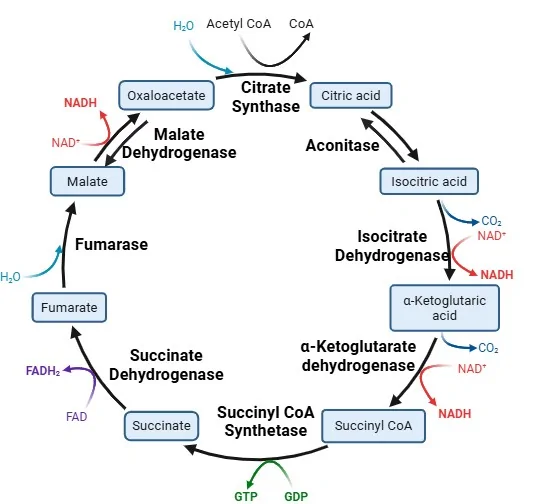
Outline the steps involved in the TCA cycle.

TCA cycle is a fundamental metabolic pathway that consists biochemical reactions interconnected in the form of a loop. The cycle acts as a central hub during cellular metabolism, as the initial metabolite in the cycle i.e. acetyl coenzyme A (CoA) generated from the breakdown of carbohydrates, fatty acids, and proteins. The next step involves isomerization of citrate into its isomeric form, isocitrate, via a reaction facilitated by aconitase (Fig. 2). Following this, two successive oxidative decarboxylation occur. In first reaction, isocitrate is converted to the five-carbon α-ketoglutarate (α-KG) in a reaction mediated by isocitrate dehydrogenase (IDH), along with NAD^+^ to NADH reduction and release of CO_2_. Further, α-KG undergoes decarboxylation byα-ketoglutarate dehydrogenase (KGDH) to form the four-carbon succinyl CoA and releases one molecule of NADH and CO_2_. Subsequently, Succinyl CoA synthetase (SCS) facilitate the generation of succinate from succinyl CoA, in a reaction coupled with the generation of GTP through substrate-level phosphorylation. It is followed by the succinate dehydrogenase (SDH) mediated conversion of succinate into fumarate, that is associated with FAD reduction to FADH2 via transfer of two electrons. Next, malate dehydrogenase (MDH) converts fumarate into malate, which is then oxidized to regenerate oxaloacetate, which combine with another acetyl-CoA molecule, ensuring the continuation of the TCA cycle and its vital role in cellular respiration.

## 3. TCA cycle and AD

### 3.1 Aconitase

A comprehensive analysis of TCA cycle-related gene expression using large-scale mRNA profiling data demonstrated that these genes show reduced expression in both AD brain tissues as well as blood, suggesting impaired TCA cycle function in the AD brain [97]. Raukase *et al*. found that aconitase activity was markedly reduced in the occipital primary cortex (OC) and frontal cortex (FC) post-mortem autopsy samples in individuals with Swedish Familial AD [98]. The lympocytes collected from AD and MCI patients displays significantly lower expression of aconitase [99]. They also observed that aconitase expression exhibits a positive co-relation with plasma vitamin E levels as well as Mini-Mental State Examination scores [99]. AD related downregulation of aconitase was further validated through an *in vivo* study in wistar rats where Aβ was injected into hippocampus to induce AD, where the aconitase activity was found to be around 40% lower [100]. Upon treatment of murine as well as cultured human neuroblastoma cells with Aβ resulted in the reversible inactivation of aconitase which preceded the neuronal death [101]. Moreover, the activity of cis aconitase decreases with the administration of aluminium [102], which is a well-established AD risk factor [103-106]. Similar results were corroborated by Mailoux*et al*, who reported the aluminium mediated down expression of aconitase along with other enzymes of TCA cycles such as IDH, SDH, KGDH and fumarase in cultured hepatocytes using western blotting [107]. Interestingly, administration of an autophagy inhibitor i.e. 3-Methyl Adenine [108] indirectly decreases the activity of aconitase via inhibition of Akt phosphorylation and aggravates cognitive impairment following Aβ injection [109]. Taken together, these evidences indicate that activity and/or expression of aconitase declines with AD and its activation can be a novel therapeutic strategy for AD.

### 3.2 Isocitrate dehydrogenase

The IDH family consists of three isozymes namely IDH1, IDH2 and IDH3, that catalyse the generation of α-ketoglutarate from isocitrate to facilitates energy oxidative phosphorylation. IDH3 is a mitochondrial heterotetrameric protein (2α, 1β and 1γ subunit) and contributes critically towards TCA cycle [110]. A significant decrease IDH3β level was observed in brain samples collected from AD patients and 5xFAD Transgenic AD mice at 9 month of age [111]. However, they did not observed any change with respect to the level of IDH3α and IDHγ [111]. However, Sang *et al.* reported a decrease in IDH3α, IDH3α and IDHγ expression [112]. The difference in the outcome between these studies can be attributable to different study population as the former was conducted in a Chinese population [111] while the later was carried out in the New Zealand [112]. Moreover the same study reported that knocking out IDH3β in 5XFAD mice resulted in lactate accumulation and increased expression of paired-box gene 6 (PAX6), that in turn silences the expression of IDH3β and exacerbate the learning and memory deficits [111]. Furthermore, a decrease in the expression of IDH3α was reported in cortex of triple transgenic mouse model of AD (3xTg-AD) using two-dimensional gel electrophoresis along with tandem mass spectrometry [113]. Metabolite profiling revealed a significant elevation of circulating isocitrate level in AD patients, suggesting a decrease or inactivation of IDH [114].

### 3.3 α-ketoglutarate dehydrogenase

αKGDH is found in mitochondria where it catalyze the oxidative decarboxylation of α-ketoglutarate to succinyl-CoA by using cofactor thiamine diphosphate (TDP) [115]. It consists of three subunits namely E1 (α-KGDH), E2 (dihydrolipoamide succinyltransferase, DLST), and E3 (dihydrolipoamide dehydrogenase, DLD) [115]. Gibson et al. reported an almost complete inhibition of enzyme activity (by 75 to 100%) of in postmortem cerebral cortex of patients with AD, while no significant alteration was observed in the peripheral tissues [116]. A subsequent study reported confirmed a marked reduction in the activity of α-ketoglutarate dehydrogenase within the temporal cortex of AD patients [117]. As described by Mastrogiacomo *et al.*, of α-KGDH enzymatic activity declines by 25-68% in AD patients when measured in the presence of exogenous TDP [118]. Moreover, their findings indicate a significant inverse relationship of α-KGDH activity with the presence of neurofibrillary tangles [118], suggesting that reduction in enzyme reflects the severity of disease. Moreover, previous studies reported that activity level of α-KGDH in AD patients with APP670/671 Swedish mutation, that results in Aβ accumulation [119-121].

The activity of α-KGDH exhibited substantial variability and did not conform to a normal distribution pattern, which can be explained in part by general instability of the enzyme following death and changes in the hypoxic state before death, lactic acid accumulation and pH [122]. Further investigations into the role of α-KGDH involvement in AD were based on protein levels estimation by western blotting in AD patients and it was observed that the concentrations of all three subunits significantly declines in the hippocampus, temporal cortex and parietal cortex [123]. Sheu and colleagues reported an additional 29-kDa protein band for E2 in the fibroblasts collected from chromosome early onset 14-linked AD [124]. However, the 29 kDa band was not present in the western blotting experiment conducted by Mastrogiacomo *et al.* [123], suggesting that extra band may represent a characteristic trait of late-onset AD. Interestingly, neurons enriched with α- α-KGDH in cortical layers III and V are more vulnerable to selective degeneration [125, 126]. A couple of studies reported the effect of DLST polymorphism on the risk of AD and it was observed that DLST A19,117, T19,183 in homozygous condition were associated with a reduced risk of AD [127, 128]. Similar findings were made by Brown *et al* in a Ashkenazi Jewish Population [129]. However, Matsushita et al. did not find any relation between AD and DLST SNPs in Japanese population [130]. Recently, Csaban *et al.* identified a rare variant 788 G > A (R263H, rs145670503) within exon 9 of gene encoding DLD in the temporal, frontal and parahippocampal lobes of an AD patient, who passed away at an age of 64 and was not found in the control population [131].

Significantly, the mechanistic contribution of α-KGDH has been thoroughly investigated in the pathogenesis of AD. Casley *et al.* demonstrated that the activity of α-KGDH was inhibited by amyloid-beta Aβ, a crucial pathological hallmark of AD [132-134]. Moreover, inhibition of α-KGDH by α-Keto-β-methyl-n-valeric acid (KMV) led to the depletion of intracellular Ca^2+^ stores by 23% upon stimulation with bradykinin [135]. Furthermore, KMV administration in N2a cells enhanced cytochrome *c* accumulation in the cytosol by 161% [135]. KMV shares structural similarity with α-ketoglutarate and inhibits α-KGDH within isolated rat brain and mitochondria without affecting pyruvate dehydrogenase [136, 137]. Additionally, α-KGDH can be inhibited by acrolein, a potent electrophilic α,β-unsaturated aldehyde formed *in vivo* through the oxidation of polyunsaturated fatty acids, such as arachidonic acid [138]. As a result, acrolein induces synaptic dysfunction and cognitive impairment via RhoA/Rho-kinase2 (ROCK2) signalling [139] and thereby used to create a sporadic AD model [140].

**Figure 3.**
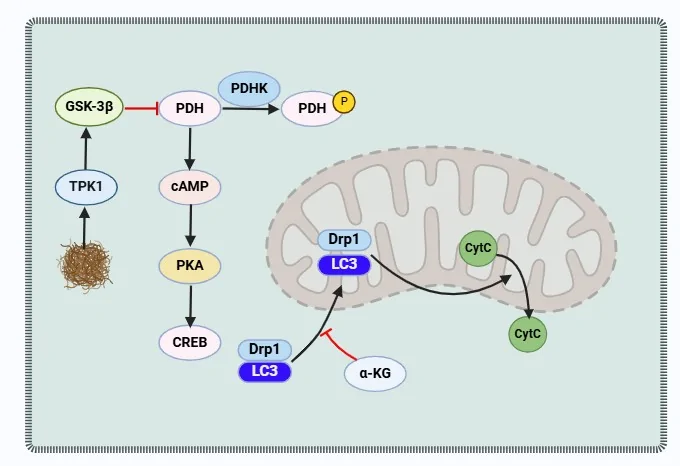
**Mechanistic role of pyruvate dehydrogenase (PDH) and α-ketoglutarate dehydrogenase (α-KGDH) in the pathogenesis of Alzheimer’s disease (AD)**. α-KGDH prevents the translocation of dynamin-related protein-1 (Drp1) and microtubule-associated protein 1A/1B-light chain 3 (LC3) from the cytosol to the mitochondria inhibits the release of cytochrome c. PDH prevents lactate accumulation via activation of cyclic AMP-protein kinase A-cAMP response element-binding protein (cAMP/PKA/CREB) pathway. Aβ can inhibits the activity of pyruvate dehydrogenase via tau protein kinase I/glycogen synthase kinase 3β (TPKI/GSK-3β). Pyruvate dehydrogenase kinase (PDHK), a serine/threonine kinase that specifically targets pyruvate dehydrogenase, negatively regulates its activity by phosphorylating the E1α subunit of the PDH complex.

Surprisingly, numerous studies have highlighted the potential therapeutic benefits of α-KGDHC short-term inhibition as a therapeutic strategy for neurodegenerative disorders, indicating its involvement as a dual-edged sword role. Carboxy ethyl ester of succinyl phosphonate (CESP) is another powerful inhibitor of α-ketoglutarate dehydrogenase, which is swiftly converted into succinyl phosphonate (SP) inside cells and specifically targets the inhibition of intracellular α-ketoglutarate dehydrogenase [141]. Administration of SH-SY5Y cells with CESP led to enhanced translocation of microtubule-associated protein 1A/1B-light chain 3 (LC3) along with dynamin-related protein-1 (Drp1) into mitochondria from the cytosol, from the cytosol leading to mitophagy followed by cytochrome C release (Fig. 3) [142]. In addition, co-administration of SP in a moderate dose along with Aβ in wistar rats within CA1 area of hippocampus preserved that spatial memory, prevented the neurodegeneration and normalised the levels of antioxidant [143].

Several reports have suggested that α-ketoglutarate dehydrogenase is particularly sensitive to the ROS, a pivotal player in the AD pathogenesis [144-149]. ROS such as peroxynitrite, NO [150], hydroxynonena l [151], hypochlorous acid, mono-N-chloramine [152] and H_2_O_2_ [153]_._ Genetic ablation of DLST promotes Aβ accumulation, along with nitrotyrosine levels, while also exacerbating memory deficits and spatial learning and in female Tg19959 mice [154], that harbors two human APP mutations namely KM670/671NL and V717F [155]. Furthermore, DSLT^+/-^ mice exhibits 80% increase in the glucose level estimated using labelled [U-^13^C] glucose, suggesting a decrease in the cortical utilisation [156]. Primary cortical neurons collected from DSLT^+/-^ mice displays higher bradykinin or caffeine releasable calcium stores when compared with wild type, a phenomena generally observed in AD [157]. Although the inhibition of DSLT was found to exacerbate the AD pathological features in murine model, DLD inhibition was protective in *Caenorhabditis elegans* model of AD. DLD inhibition by 2-methoxyindole-5-carboxylic acid (MICA) prevents decreases oxidative stress and oligomerization of Aβ using *C. elegans* transgenic strains CL2006 and CL4176, which human Aβ_42_ peptide expression[158]. Furthermore, RNAi mediated suppression or MICA based inhibition of DLD significantly attenuates phosphorylation of tau by enhancing glucose uptake in *C. elegans* transgenic strain VH255 hdEx82 that expresses human fetal 352aa CNS tau [159].

### 3.4 Succinyl CoA synthetase

Succinyl CoA synthetase (SCS) facilitates succinyl CoA conversion into succinate, representing the only step involves substrate-level phosphorylation of either ADP or GDP [160]. Eukaryotic SCS is a heterodimer consisting of two subunits namely α and β, with the former being the catalytic subunit. β subunit exist in two isoforms i.e. GTP-forming SUCLG and ATP-generating SUCLA2 [161, 162]. Recently, Jia *et al.* reported that the level of SUCLA2 significantly declinies in AD brain and blood cells using an integrated transcriptomic approach [163]. Furthermore, they reported that the levels of SUCLA2 correlated negatively with Aβ and tau levels, while a positive correlation exists with MMSE score [163]. Conditional knockout of SUCLA2 in the forebrain of mice leads to increased succinylation, that in turn results in respiratory chain complex I activity reduction accompanied by widescale changes in gene expression [164]. Notably, a previous study reported that alteration in the protein succinylation signatures correlated well with changes in terms of metabolic function within AD brain [165]. While SCS itself has not been directly implicated as a primary target in AD, disruptions in the broader metabolic pathways involving SCS may contribute to the disease's progression.

### 3.5 Succinate dehydrogenase (SDH)

SDH complex converts succinate into fumarate, while also transferring electrons to the ubiquinone (UQ) pool of the respiratory chain. SDH consists of 4 subunits, of which A and B forms the *sensu stricto,* while C and D are reuired during the electron transfer from succinate to the UQ pool [166]. Involvement of SDH in AD was reported for the first time by Kaneko and colleagues, where they described that Aβ_25-35_, its d-Ser^26^-substituted derivative, and Aβ_1-40_ can inhibit SDH within Hela cells using MTT [3-(4,5-dimethylthiazol-2-yl)-2,5-diphenyltetrazolium bromide] [167]. Subsequently, Abe and colleagues contradicted these results, indicating that Aβ reduces cellular MTT reduction [168]. The observed discrepancy is attributable to Aβ peptide treatment inducing enhanced exocytosis of the MTT dye, thereby reducing its intracellular retention and subsequent conversion into formazan crystals [168]. Additionally, unlike other TCA cycle enzymes, the activity of SDH is elevated following aluminium administration [102], which is known to increase the risk of AD [103-106]. Similarly, SDH expression was found to be elevated within SH-SY5Y cells following Aβ treatment, an effect that was reduced by the mutated AβY10A, indicating that tyrosine residue at 10^th^ position is critical for SDH alterations. [169]. Collectively, these findings suggest that SDH inhibition can be explored as novel therapeutic approach for AD treatment. However, induction of AD like symptoms using intracerebroventricular-streptozotocin (ICV-STZ) in Sprague-Dawley (SD) male rats resulted in decreased in the activity of SDH activity [170]. Additionally, it was observed that administration with trans,trans-Farnesol (TF) improved the cognitive function following ICV-STZ by normalisation of the SDH activity level [170].

### 3.6 Fumarase

In human cells, the fumarase is encoded by FUM1 gene that consist of multiple transcription sites giving rise to either a full-length protein that comprises of fumarase mitochondrial targeting sequence (MTS) necessary for mitochondrial targeting and the truncated form that lacks it and stays within cytosol [171-174]. Bubber *et al* reported that activity level of fumarase did not changes within the postmortem brain collected from the AD patients [175]. However, in contrast, a recent report suggested that fumarase transcript level declines with AD, suggesting the need for further investigation [163].

### 3.7 Malate dehydrogenase (MDH)

MDH belongs to a group of protein called as nicotinamide adenine dinucleotide (NAD)-dependent dehydrogenases or oxidoreductases [176, 177] and generates oxaloacetate from malate by using NAD+/NADH as a cofactor [178-180]. MDH primarily exists in two isoforms: MDH1 that’s present in cytosol and plays a pivotal role in malate/aspartate shuttle and MDH2 which converts malate to oxaloacetate in mitochondria [181, 182]. Using label free quantitative proteomic analyses and MRM-based triple quadrupole mass spectral assay based target groups, two independent studies reported an elevation in the level MDH within cerebrospinal fluid collected from AD patients [183, 184]. Furthermore, Elevated MDH2 enzymatic activity was observed in brain tissue obtained from AD patients [175, 185]. In contrast, the MDH1 gene was observed to be downregulated in AD through integrated analysis of transcriptomic data [163]. Shi *et al* proposed a possible mechanism showing that MDH2 mRNA can be silenced by miR-743a, which in turn decreases in response to increased oxidative stress [186], a crucial player in AD pathophysiololgy [187-194]. In contrast, MDH was found to be downregulated in experimental models of AD, indicating the differences between molecular data obtained from or postmortem tissue and *in vivo* studies, which could be influenced by factors such as disease stage, tissue-specific expression, or compensatory mechanisms in response to neurodegeneration [195, 196]. In addition, post-mortem delay can affect enzyme integrity and activity, thereby confounding results and can mask the actual effects. Furthermore, region specificity further complicates interpretations, since distinct areas (e.g., the hippocampus versus the cerebellum) exhibit unique metabolic demands and vulnerabilities that result in varied enzyme expression and activity profiles. Together, these factors necessitate rigorous methodological controls and interpretation of postmortem data to accurately elucidate the molecular pathways contributing towards AD.

Beyond this overview, one might also consider how emerging imaging techniques and biomarker studies are addressing these challenges, offering more refined temporal and spatial resolution in tracking enzyme dynamics.

### 3.8 Citrate Synthase (CS)

CS catalyze citrate formation via condensation of oxaloacetate and acetate residue of acetyl-CoA [197, 198]. The citrate thus synthesised is further metabolized to produce 2 NADH, 1 FADH2, and 1 ATP, subsequently utilized in electron transport chain (ETC) and generate additional ATP through oxidative phosphorylation, facilitated by cytochrome oxidase enzymes (complex IV) in the mitochondria [199]. Jia *et al.* identified a significant reduction in CS expression with AD, suggesting a potential impairment in mitochondrial function, which is essential for cellular energy production [163]. Subsequent reduction in the ATP synthesis creates a low energy environment in the brain [200] that impair normal function of cellular processes, including protein clearance mechanisms, thereby allowing Aβ to accumulate and aggregate [201-204]. Moreover, under conditions of reduced ATP availability, mitochondria are unable to expel Ca^2+^ from the inner mitochondrial membrane, which consequently leads to mitochondrial dysfunction, impaired mitophagy, and neuronal cell death [205-209]. Thereafter, citrate-malate antiporter expels the citrate citrate into the neuronal cytosol from mitochondria [210-213]. Once in the cytoplasm, enzyme ATP citrate lyase converts citrate into acetyl-CoA by the [214, 215]. Acetyl-CoA then combines with choline to synthesize acetylcholine, a process catalyzed by the enzyme choline acetyltransferase [216-218]. Acetylcholine is a critical neurotransmitter involved in various cognitive functions, particularly memory [219, 220]. Its deficiency is a hallmark of AD, contributing to the cognitive decline observed in affected individuals [221-224].

### 3.9 Pyruvate dehydrogenase (PDH)

Pyruvate undergoes oxidative decarboxylation by PDH and generate acetyl-coenzyme A, thereby acting as a fundamental metabolic bridge connecting glycolysis to the citric acid cycle [199, 225]. Pyruvate dehydrogenase consists of three subunits namely: E1 (pyruvate dehydrogenase), E2 (dihydrolipoyltransacetylase), and E3 (dihydrolipoyl dehydrogenase) [226-228]. These enzymes work in concert to convert pyruvate into acetyl-CoA. E1 carries out the decarboxylation of pyruvate, while E2 transfer acetyl group to Coenzyme A, and E3 regenerates the oxidized form of lipoamide cofactor, ensuring the continuation of the reaction cycle [226-228]. Sorbi and colleagues reported a decline in the activity of PDH within frontal cortex of AD patients and correlated with low choline acetyltransferase activity [229]. Other studies have also reported a comparable reduction in the PDH activity with AD, suggesting a consistent impairment, which could be linked to disruptions in cellular metabolism [230, 231]. In line with clinical studies, Ding *et al* found that an age-related decline in E1 activity within the hippocampus female 3xTgAD mice [232], as determined by the estimation of an inhibitory phosphorylation site at Ser293 [233]. In contrast to the 3xTgAD mice, the activities of E1 was found to be unaffected in mitochondria and synaptosomes of Tg2576 mice, suggesting a compensatory mechanisms that help maintain cellular function in the face of neurodegenerative processes [234].

Chen *et al* generated a hippocampus specific conditional PDH knockout mice using Cre-Lox strategy and observed that *Pdh^-/-^* Knockout mice exhibits learning impairment and lactate accumulation via inhibition of cyclic AMP-protein kinase A-cAMP response element-binding protein (cAMP/PKA/CREB) pathway [235]. 3-Bromopyruvate, a suicide inhibitor of PDH complex, induces long-term learning deficits, suggesting the protective role of enzyme complex [236]. Interestingly, chronic low-level exposure of lead may elevate AD risk through several mechanisms [237-245], one of which involves the direct inhibition of PDH [246]. In fact, Aβ can inhibits the activity of PDH via tau protein kinase I/glycogen synthase kinase 3β (TPKI/GSK-3β), results in mitochondrial impairment and contribute towards AD related energy deficits [247, 248]. Acrolein, as previously discussed, is known to inhibit the activity of α-KGDH, an essential enzyme in TCA cycle. Additionally, acrolein has also been shown to interfere with the function of pyruvate dehydrogenase and potentially contribute towards the progression of AD [249]. Regulating the upstream pathway of pyruvate dehydrogenase also possess the potential has the potential to attenuate AD pathology, thereby improving mitochondrial function and reducing neurodegenerative processes associated with the disease. For instance, pyruvate dehydrogenase kinase (PDHK), a serine/threonine kinase that specifically targets pyruvate dehydrogenase, negatively regulates its activity by phosphorylating the E1 subunit [250]. A novel inhibitor of PDHK i.e. compound A was able to improve cognitive function, as demonstrated by performance in the Morris water maze and novel object recognition test in 5XFAD mice [251]. Additionally, Compound A attenuates neuronal death in the cerebral cortex and hippocampus and, without influencing Aβ deposition [251].

The TCA cycle, crucial for cellular energy production, is disrupted in AD, with several enzymes involved in the cycle showing altered expression and activity in AD patients, cell culture models and animal models. These alterations are linked to mitochondrial dysfunction, energy deficits, and Aβ accumulation. Collectively, these findings underscore the pivotal role of TCA cycle enzymes in the AD pathogenesis, implicating mitochondrial dysfunction and metabolic disturbances as key contributors to cognitive decline and neurodegeneration. Thereby, in the next section we will discuss various small molecule activator and inhibitors for TCA cycle that can be further exploited as possible therapeutic interventions in the future.

## 4. Modulators of TCA cycle components

In the current section, we will explore a range of small molecule activators and inhibitors of the TCA cycle, highlighting their potential as promising therapeutic agents. These compounds which are summarised in the Table 1, which modulate the activity of key enzymes within the TCA cycle, offer opportunities to correct the metabolic dysfunctions observed in AD. For IDH1, inhibitors such as AGI-5198, ML309, AG-120, GSK321 and Compound 1 have been developed specifically for R132H mutant, which is known to be associated with acute myeloid leukemia [252-254], glioblastoma [255-257], intrahepatic cholangiocarcinoma [258] and central/periosteal chondrosarcoma [259]. AGI-5198 was initially identified as compound 35 in a high-throughput screen for compounds that inhibit the IDH1-R132H mutant homodimer. The compound exhibits potent inhibitory activity against R132H mutant with an IC_50_ value of 0.07 µM [260, 261]. The same studies further reported rapid turnover in human and rat with an estimated hepatic extraction ratio of 0.93 and 0.85, respectively. Reasonable plasma exposure was achieved via intraperitoneal dosing at 50 mg/kg with an AUC0-24h value of 20800 h·ng/mL), enabling the use of AGI-5198 for further *in vivo* studies. AGI-5198 was further derivitise to yield a racemic mixture of (-)- and (+)-2-(2-(1H-Benzo[d]imidazol-1-yl)-N-(3-fluorophenyl)acetamido)-N-cyclopentyl-2-o-tolylacetamide ((-)-ML309 and (+)-ML309), that inhibits R132H mutant with an IC_50_ value of 96 nM [262]. ML309 was found to be relatively stable in the human plasma with a half-life of around 3 hours in mice as determined by PK studies [262]. Another derivative of AGI-5198, known as AG-120 (ivosidenib), demonstrated enhanced potency against the R132H mutant, with an IC_50_ value of 12 nM [263]. At a recommended dose of 50mg of AG-120, majority of the adverse effects belongs to grade 1 and grade 2, with the most common being diarrhea, fatigue, and nausea [264]. The recommended dose was sufficient to clear the burden R132H mutation as determined variant allele frequency (VAF) analysis using next-generation sequencing (NGS) [264]. Collectively, these findings substantiate ivosidenib's favorable clinical safety profile and support its advancement into further clinical evaluations. Together these points established a safe clinical profile of ivosidenib for clinical studies. Administration of ivosidenib along with azacitidine resulted in a significant clinical outcome with median survival for ivosidenib/azacitidine and azacitidine were 24 and 7.9 months respectively [265]. A high-throughput biochemical screen targeting led to the discovery of a series of tetrahydropyrazolopyridine (THPP) inhibitors targeting an IDH1 heterodimer—composed of the R132H mutant [266]. Further substitution on the aniline ring and modification of the THPP core with (R)-7-methyl resulted in the identification of GSK321 as a highly potent inhibitor of mutant R132H IDH1 enzymes with IC_50_ values of 4.6 nM [266]. Nonetheless, the efficacy and safety of GSK321 still being studied in the preclinical stage and has not yet advanced to clinical trials [267]. Using high throughput screening, Deng *et al* identified compound 1 as a novel allosteric inhibitor of mutant R132H IDH1 with an IC50 value of 81.5 nM [268]. Compound 1 interacts with Asp^279^ to prevent its interaction with Mg^2+^[268], a step essential for the catalytic activity of the enzyme [269]. Although, R132H mutation has been studied extensively in haematological malignancies, the exact biological role and pathogenic impact of the R132H mutation in AD remain unexplored, thereby opening a novel mechanistic domain for exploration.

**Table 1:** Describes activators and inhibitors for TCA cycle enzymes.

Enzyme	Compound	Inhibitors/ Activators	Efficacy	Reference
Isocitrate dehydrogenase (R132H)	AGI-5198	Inhibitors	IC_50_= 0.07 µM	[260, 261]
	ML309	Inhibitors	IC_50_= 96 nM	[262]
	AG-120	Inhibitors	IC_50_= 12 nM IC_50_= 8 nM in Caco2 cells	[263]
	GSK321	Inhibitors	IC_50_= 4.6 nM	[266]
	Compound 1	Inhibitors	IC_50_= 81.5 nM	[268]
α-Ketoglutarate dehydrogenease	(S)-2-[(2,6-dichlorobenzoyl) amino] succinic acid (AA6)	Inhibitors	IC_50_= 5.1 mM	[270] [271]
	SP (succinyl phosphonate) (phosphonoethyl SP (PESP) carboxyethyl SP (CESP)	Inhibitors	100% inhibition at 0.01 mM 70% inhibition at 0.01 mM in cultured fibroblasts	[277]
Succinate dehydrogenase	N-methoxy-(biphenyl-ethyl)-pyrazole-carboxamide derivative	Inhibitors	IC_50_= 14 nM	[281]
	Compund 12 *[N*-(4-fluoro-2-(phenylamino)phenyl)-pyrazole-4-carboxamide)]	Inhibitors	IC_50_ = 1.836 mg/L	[282]
Fumarase	compound 3	Inhibitors	Ki= 4.5 µM	[283]
	5-(4-chlorophenyl)-1,3-dimethyl-6-pentyl-1,6-dihydro-2H-pyrrolo[3,4-d]pyrimidine-2,4(3H)-dione	Activator	AC_50_=5.6 µM	[284]
Malate dehydrogenase	methyl 3-(3-(4-(2,4,4-trimethylpentan-2-yl)phenoxy)propanamido)benzoate	Inhibitors	IC_50_= 1.06 µM	[285]
	(E)-4-((4,6-dimethylpyrimidin-2-ylthio)methyl)-N'-(1-(4-methyl-3-nitrophenyl)ethylidene)benzohydrazide	Inhibitors	IC_50_= 3.9 µM	[297]
Citrate synthase	fluorovinyl thioether	Inhibitors	K_i_=4.3βμm	[287]
	butyryl-CoA octanoyl-CoA palmitoyl-CoA	Inhibitors	IC_50_= 640 µM IC_50_= 436 µM IC_50_= 340 µM	[288] [288] [288]
	Spermine*	Activator	AC_50_= 50 µM	[289]
Pyruvate dehydrogenase	Hydroxamate	Inhibitors	IC_50_= 3.8 µM	[295]
	Compound 33	Inhibitors	IC_50_= 2.5 µM	[296]

Another critical enzyme in the TCA cycle, α-KGDH, has been a primary target in the development of various inhibitors. The compound (S)-2-[(2,6-dichlorobenzoyl) amino]succinic acid (AA6) was originally found to elevate α-KG levels in cardiac mesenchymal cells isolated from diabetic mice, leading to the hypothesis that AA6 might inhibit α-KGDH activity [270]. However, subsequent research by Blua *et al.* refuted this notion, demonstrating that the AA6-induced increase in α-KG levels was not attributable to inhibition of α-KGDH due to relatively higher IC_50_ value [271]. Another class of α-KGDH inhibitors includes the substitution of carboxyl group of α-keto acids with a phosphonate, which in turn targets the enzyme-bound ThDP [272-276]. Subsequently, Bunik *et al* reported that phosphonate analogue of α-KG i.e. succinyl phosphonate (SP) and its phosphonoethyl (PESP) and carboxyethyl (CESP) esters resulted in the 100% and 70% inhibition of purified and cellular α-KGDH respectively at a concentration of 10 µM [277].

Several inhibitors have been developed against SDH and majority of them were derived from pyrazol-5-yl-benzamide through scaffold hopping and reversing the direction of amide groups [278-280]. The most potent of them were N-methoxy-(biphenyl-ethyl)-pyrazole-carbo-xamide, which were designed on the basis of based on the basis of fluxapyroxad and pydiflumetofen binding mode [281]. Among them, compound 7s exhibited an IC_50_ value of 0.014 µM, which is about 200 fold potent than fluxapyroxad (IC50 = 2.88 μM) [281]. A novel class of SDH inhibitors comprises a series of N-(4-fluoro-2-(phenylamino)phenyl)-pyrazole-4-carboxamides synthesized via scaffold hopping from bixafen, among which compound-12 demonstrated the highest potency with an IC_50_ of 1.836 mg/L and robust binding interactions as confirmed by molecular modelling [282].

Compound 3 was identified as a novel fumarase inhibitor through high-throughput screening of nutrient-dependent cytotoxic compounds, followed by target identification utilizing a photoaffinity labeling strategy [283]. In cultured SW620 cells, pyrrolidinone-based compound 1 exhibited cytotoxic activity and was subsequently modified to yield compound 2 by truncating the propargyl moiety to a methyl group [283]. The ethyl ester derivative, compound 2, was then converted into the corresponding carboxylic acid, compound 3, which inhibited fumarase activity in a dose-dependent manner, with a Ki value of 4.5 µM [283]. Zhu et al. screened six in-house small-molecule libraries comprising a total of 57,037 compounds and identified a series of phenyl-pyrrolo-pyrimidine-diones as fumarase activators. Among these, the compound 5-(4-chlorophenyl)-1,3-dimethyl-6-pentyl-1,6-dihydro-2H-pyrrolo[3,4-d]pyri-midine-2,4(3H)-dione emerged as the most potent, with an AC1.5 value of 5.6 µM [284].

Naik et al. synthesized a series of novel trimethylpentane derivatives demonstrating dual inhibitory activity against MDH1 and MDH2. Utilizing structure-activity relationship (SAR) studies, they found that the compound methyl 3-(3-(4-(2,4,4-trimethylpentan-2-yl)phenoxy)propanamido)benzoate inhibited MDH1 and MDH2 with IC_50_ values of 1.07 and 1.06 µM, respectively, and subsequently exhibited antitumor activity in a mouse xenograft model [285]. Similarly, (E)-4-((4,6-dimethylpyrimidin-2-ylthio)methyl)-N'-(1-(4-methyl-3-nitrophenyl)ethylidene)benzohydrazide was identified as an MDH2 inhibitor based on its structural similarity to the known inhibitor LW6 [286].

Both inhibitors and activators have been developed for citrate synthase. For example, inhibitors for citrate synthase were synthesised by mimicking the enolate intermediate in the enzyme reaction [287]. The most potent inhibitor, compound 9 fluorovinyl thioether exhibited a Ki of 4.3 μM, wherein a fluorine replaces the oxygen atom of the enolate. In contrast, the non-fluorinated vinyl thioether analogue 10 showed a Ki of 68.3 μM, clearly demonstrating the role of fluorine in the inhibition [287]. Notably, fatty acyl-CoAs—specifically butyryl-CoA, acyl-CoA, and palmitoyl-CoA—exhibit inhibitory effects on citrate synthase with IC_50_ values of 640 µM, 436 µM, and 340 µM, respectively [288]. These relatively high IC_50_ values suggest that these compounds are unlikely to have significant pathophysiological or therapeutic impacts. On the other hand, citrate synthase activity can be enhanced by spermine with an AC_50_ value of 50 µM [289], which belongs to the low molecular weight aliphatic amines also referred to as polyamines [290-292]. Notably, the administration of spermine in wister rat and gerbils exhibits a significant neuroprotective effect upon hypoxia- ischemia based injury [293, 294].

PDH is a central regulator of cellular metabolism, with its E1 subunit (PDH E1) plays a crucial role by catalyzing the conversion of the cofactor thiamine pyrophosphate (TPP) into a reactive intermediate, which then facilitates the reductive acetylation of the lipoamide moiety on the E2 subunit. Recent advancements have focused on the development of furan-based thiamine analogues designed to interfere with PDH E1 activity. Notably, hydroxamate derivative [295] and compound 33 [296] have been synthesized, displaying inhibitory effects on PDH E1 with IC_50_ values of 3.8 µM and 2.5 µM, respectively. Engineered to mimic essential features of the TPP cofactor, these analogues effectively disrupt the normal enzymatic function of PDH E1, thereby impeding the formation of the reactive intermediate necessary for acetylating lipoamide.

## Conclusion

TCA cycle is a central metabolic pathway that promotes neuronal function by providing energy for various cellular processes, particularly in the brain that exhibit a significant higher demand of energy. Disruption of key TCA cycle enzymes promotes the mitochondrial dysfunction, ATP depletion, and the accumulation of toxic metabolites, that exacerbates the pathology of AD. Disruption of the TCA cycle initiates a cascade of deleterious events—inducing oxidative stress, neuroinflammation, and neuronal injury—which together establish a self-perpetuating cycle that accelerates AD progression. Moreover, impaired TCA function reduces mitochondrial NADH and ATP synthesis, undermining cellular metabolism and stress responses that facilitate Aβ accumulation and tau hyperphosphorylation. Moreover, this metabolic failure disrupts redox homeostasis by impairing key TCA enzymes—particularly those with iron-sulfur clusters—thereby elevating oxidative stress and further promoting the neurodegeneration. Interestingly, modulation of TCA cycle enzymes, through either activation or inhibition, holds promise as a targeted approach to restore metabolic balance within the brain. While inhibitors and activators of TCA cycle enzymes have been extensively studied in cancer research for their potential to modulate tumour metabolism, their effects in the AD models remain underexplored and warrant further investigation to determine their therapeutic potential.
